# Clinical and Biochemical Characteristics of Hemophagocytic Lymphohistiocytosis in People Living With HIV and Disseminated Histoplasmosis at a Tertiary Hospital in Mexico

**DOI:** 10.1093/ofid/ofae385

**Published:** 2024-07-08

**Authors:** Arlen Cruz-Quezada, Joaquín Moreno, Miguel Ángel Solís-Bravo, Claudia Andrea López Chávez, Tiburcio Santos, Juan José Fonseca-Mata, Javier Araiza, Alexandro Bonifaz

**Affiliations:** Department of Infectious Diseases, Hospital Regional San Juan de Dios, Estelí, Nicaragua; Department of Infectious Diseases, Hospital General de México “Dr. Eduardo Liceaga,” Ciudad de México; Department of Infectious Diseases, Hospital General de México “Dr. Eduardo Liceaga,” Ciudad de México; Department of Infectious Diseases, Hospital General de México “Dr. Eduardo Liceaga,” Ciudad de México; Department of Infectious Diseases, Hospital General de México “Dr. Eduardo Liceaga,” Ciudad de México; Department of Infectious Diseases, Hospital General de México “Dr. Eduardo Liceaga,” Ciudad de México; Department of Mycology, Hospital General de México “Dr. Eduardo Liceaga,” Ciudad de México, México; Department of Mycology, Hospital General de México “Dr. Eduardo Liceaga,” Ciudad de México, México

## Abstract

**Background:**

Hemophagocytic lymphohistiocytosis (HLH) is considered a rare disease with high morbidity and mortality risks. Most research on this disease is conducted in pediatric settings. Therefore, this study aimed to describe the clinical characteristics, laboratory findings, and outcomes related to HLH in people living with human immunodeficiency virus (HIV)/AIDS) and disseminated histoplasmosis.

**Methods:**

A retrospective and descriptive study was conducted in a tertiary hospital in México City from January 2018 to December 2022, including people living with HIV who had disseminated histoplasmosis confirmed through direct microbiological or immunological methods with an HScore ≥169 or who met 5 of the 8 HLH-2004 criteria.

**Results:**

HLH occurred in 36.1% (n = 26) of patients with HIV and disseminated histoplasmosis; the majority were men (84.9%), and their mean age (standard deviation) was 30.19 (5.6) years. The most frequent clinical manifestations were hepatomegaly (100%), fever (96.2%), and dyspnea (84.6%). The most common biochemical changes were hyperferritinemia (100%), elevated lactate dehydrogenase (100%), and bicytopenia (61.5%). Partial thromboplastin time (*P* = .012) and prothrombin time (*P* = .004) were associated with the 30-day mortality rate, and the 30-day survival rate was 65.4%.

**Conclusions:**

We detected a high frequency of HLH; therefore, we encourage physicians to use diagnostic prediction tools (HLH-2004 and HScore criteria) in each reassessment for timely detection.

## Teaser

Hemophagocytic lymphohistiocytosis in people living with HIV and disseminated histoplasmosis is considered rare, and it is potentially fatal. In this review of clinical and biochemical findings, partial thromboplastin time and prothrombin time were associated with 30-day mortality rates.

Hemophagocytic lymphohistiocytosis (HLH) is a clinical entity characterized by immune dysregulation, a hyperinflammatory response associated with cytokine storm and T- and natural killer (NK) cell dysfunction, which is potentially fatal [1–3]. The Histiocyte Society has classified HLH as primary and secondary: primary HLH is observed mainly in the pediatric population and is frequently caused by genetic mutations, whereas secondary HLH is triggered by infections, neoplasms, or autoimmune diseases [4–7]. However, the North American Consortium for Histiocytosis does not recommend this classification, as overlapping entities may exist [4]. Due to limited information and underdiagnosis, it is difficult to accurately estimate the frequency of HLH. Case series and retrospective cohorts have been reported only with small populations worldwide, being it rare [2, 5].

It is complex to determine the main trigger of HLH in people living with human immunodeficiency virus (HIV; PLHIV) due to the overlap in pathological conditions. HLH may be secondary to acute or chronic HIV infection, immune reconstitution inflammatory syndrome (IRIS), opportunistic infections, neoplasms, or autoimmune diseases [8].

The diagnosis of HLH is challenging due to the wide spectrum of clinical presentations. The diagnostic criteria proposed by the International HLH Society in 2004 [5] include fever, splenomegaly, pancytopenia, or bicytopenia (hemoglobin <9 g/dL, absolute neutrophil count <1000/µL, platelet count <100 000/µL), hypertriglyceridemia (triglycerides >265 mg/dL), or hypofibrinogenemia (fibrinogen <150 mg/dL), hyperferritinemia (ferritin >500 ng/mL), hemophagocytosis (at bone marrow, spleen, lymph node or liver biopsy), NK cell activity (decreased or absent), and soluble CD25 (interleukin 2 >2400 U/mL). To establish the diagnosis of HLH, ≥5 of these 8 criteria must be present. Although the criteria were developed for the pediatric population, they currently constitute the basis of diagnosis in adults; however, their application has not yet been validated [4, 6, 9].

HScore is a risk stratification tool for HLH validated in the adult population [10]. It translates a probability that ranges from 0% to >99% and uses clinical (known underlying immunosuppression, temperature >38.4°C, organomegaly), biochemical (cytopenia, ferritin >2000µg/L, triglyceride >132.7 mg/dL, fibrinogen >250 mg/dL, aspartate aminotransferase ≥30 U/L), and cytological (hemophagocytosis features in bone marrow aspirate) variables. Scores ≤90 are associated with a <1% probability of HLH, while scores ≥250 indicate a >99% probability. A cutoff score of 169 corresponds to a sensitivity of 93% and a specificity of 86% [10]. Despite treatment, HLH is associated with a poor prognosis, with mortality rates of 40%–50% [9, 11–13]. There is little published information on HLH in PLHIV and disseminated histoplasmosis; therefore, our objective was to describe its clinical and biochemical characteristics and related outcomes.

## METHOD

### Study Design

A retrospective and observational study was conducted at a single center. We sought information on patients with a diagnosis of disseminated histoplasmosis in PLHIV with a first episode of HLH who were treated in the infectious diseases department of the Hospital General de México “Dr. Eduardo Liceaga” from January 2018 to December 2022.

Our study included the medical records of adults >18 years of age with a confirmed diagnosis of HIV infection and disseminated histoplasmosis who met the following inclusion criteria: HScore ≥169 and/or 5 of the 8 HLH-2004 diagnostic criteria. Since our center could not measure NK cell activity and serum levels of the soluble receptor CD25, patients had to meet the remaining criteria. Records of patients with neoplasia or autoimmune disease, pregnant women, or patients with incomplete data were excluded due to the rarity of the disease and convenience sampling. Information was entered into a data collection form that integrated sociodemographic, clinical, and biochemical variables, HScore, fungal culture, urinary antigen for *Histoplasma capsulatum*, comorbid conditions, and outcomes.

### Statistical Analysis

Statistical analysis was carried out using the IBM SPSS Statistics software (version 27). The data was classified based on the type of variables. For qualitative variables the frequency and percentage had been reported, while for quantitative variables the mean and standard deviation (SD) or median and interquartile range had been reported based on their distribution. The Shapiro-Wilk test was performed to verify normality in the quantitative variables; χ^2^ and Fisher exact tests were also conducted. Differences were considered significant at *P* < .05. Odds ratios was calculated for the risk measures, and the analysis of quantitative variables was performed with Student *t* and Mann-Whitney *U* tests. The protocol was approved by the Ethics Committee of Hospital General de México “Dr. Eduardo Liceaga,” according to Mexican research guidelines.

## RESULTS

**Figure 1.**
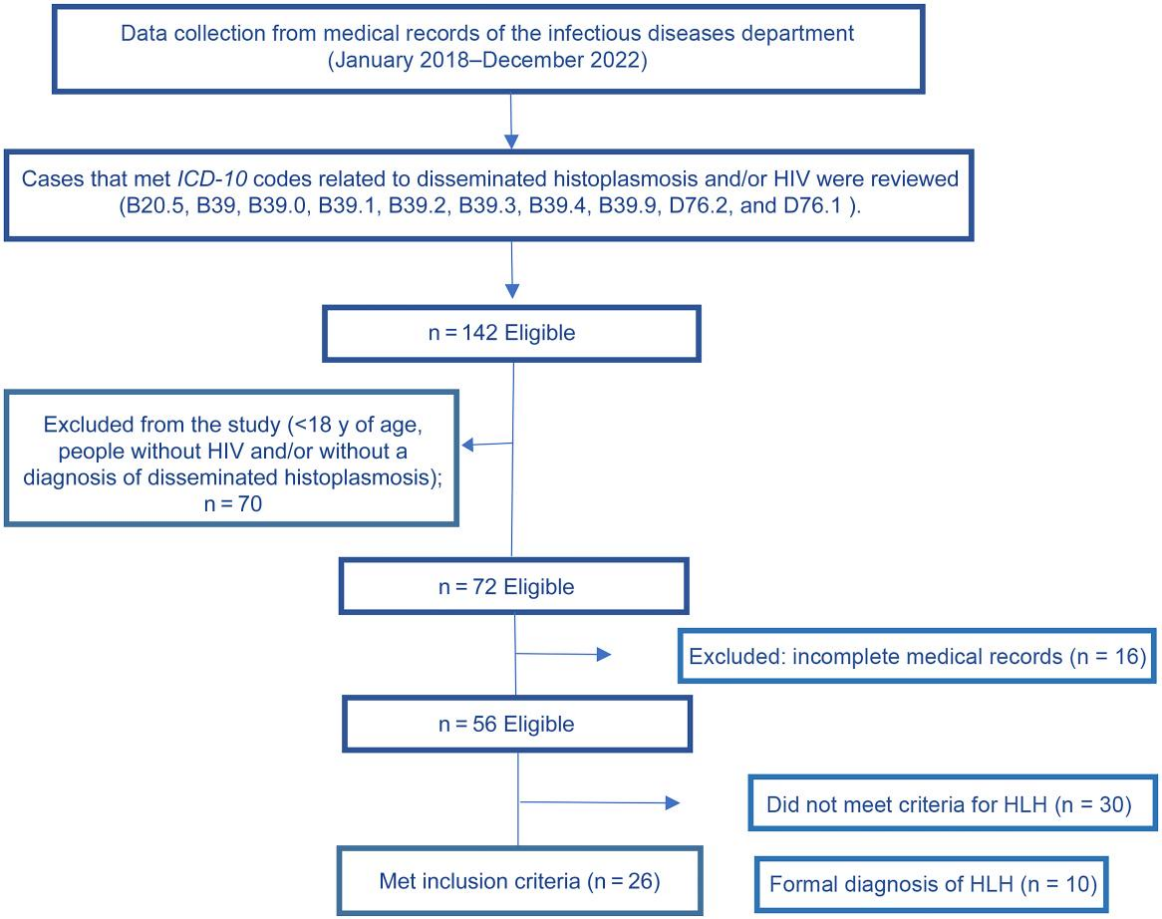
Flowchart of the medical records screening process for people living with human immunodeficiency virus (HIV), disseminated histoplasmosis, and hemophagocytic lymphohistiocytosis (HLH). Patients without an HIV diagnosis and/or with nondisseminated histoplasmosis were excluded. Abbreviation: *ICD-10*, *International Classification of Diseases, Tenth Revision.*

In total, 142 records of disseminated histoplasmosis diagnoses (according to the *International Classification of Diseases, Tenth Revision*) were evaluated; of these, only 26 met the selection criteria (Figure 1). It is possible that some patients were not seen by the infectious diseases department, which could represent selection bias, but all records of histoplasmosis reported by the mycology laboratory and all records of the informatics department were included.

### Patient Characteristics

Of 72 individuals with disseminated histoplasmosis and HIV, 26 (36.1%) met the diagnostic criteria for HLH. The data analysis revealed a predominance of men (84.6%), with a male-female ratio of 5.5:1. The mean age (SD) was 30.19 (5.6) years. Only 2 patients originated from Venezuela, and El Salvador accounted for 7.7%.

### Diagnosis of HIV and Disseminated Histoplasmosis

HIV infection was diagnosed in 50% of patients at the time of hospitalization, and the remainder knew their serological status before the presentation of HLH. Of this group, 34.6% had abandoned antiretroviral therapy (ART), and 15.4% of patients (n = 4 of 13) were identified with recent ART initiation. Furthermore, 100% of patients had severe immunosuppression, with a mean CD4^+^ T-cell count (SD) of 26/µL (20.4/µL). Disseminated histoplasmosis was confirmed in 80.7% (n = 21 of 26) through cultures, and the rest were confirmed by urinary antigen for *H capsulatum* (n = 12 of 12) and lymph node histopathology (n = 5 of 13). In addition, 100% of patients obtained an HScore >169, with a mean (SD) of 248.9 (29.1) points, of whom 34.6% met 5 of the 8 HLH-2004 criteria. Only 2 patients presented with hemophagocytosis in their bone marrow.

### Clinical and Laboratory Manifestations

The most frequent clinical manifestations were hepatomegaly (100%), fever (96.2%), dyspnea (84.6%), and splenomegaly (76.9%). The most common biochemical changes were ferritin >500 µg/L (100%), bicytopenia (61.5%), and pancytopenia (26.9%) (Table 1). The means of the biochemical parameters included or not included in the diagnostic prediction tools are shown in Table 2.

**Table 1.** Patient Characteristics and Clinical/Laboratory Results

Characteristic	Patients, No. (%)^a^ (n = 26)
Age, mean (SD), y	30.19 (5.6)
Male sex	22 (84.9)
5 of 8 HLH-2004 criteria	9 (34.6)
HScore, mean (SD)	248.92 (29.1)
Fever	25 (96.1)
Dyspnea	22 (84.6)
Hemorrhagic manifestations	3 (11.5)
Splenomegaly	20 (76.9)
Hepatomegaly	26 (100)
Bicytopenia	16 (61.5)
Pancytopenia	7 (26.9)
Ferritin >500 (µg/L)	26 (100)
Elevated lactate dehydrogenase^b^	26 (100)
Hypertriglyceridemia^c^	10 (38.4)
Hypofibrinogenemia^d^	5 (19.2)

Abbreviations: HLH, hemophagocytic lymphohistiocytosis; SD, standard deviation.

^a^Data represent no. (%) of patients unless otherwise specified.

^b^Normal value: 140-271 IU/L.

^c^Defined as triglycerides >265 mg/dL.

^d^Defined as fibrinogen <150 mg/dL.

**Table 2.** Baseline Human Immunodeficiency Virus Status and Laboratory Results

Laboratory Parameter	Mean Value (SD) (n = 26)
Viral load, copies	2 873 695.58 (4 521 829.9)
CD4**^+^** T-cell count, cells/µL	26 (20.4)
Alanine aminotransferase, IU/L	68 (47)
Aspartate aminotransferase, IU/L	252.73 (300.1)
γ-Glutamyl transferase, IU/L	268.12 (233)
Alkaline phosphatase, IU/L	592.31 (515.16)
Total bilirubin, mg/dL	1.84 (2.5)
Lactate dehydrogenase, IU/L	2051.69 (2826)
C-reactive protein, mg/L	201.73 (99.8)
Triglycerides, mg/dL	300.73 (243.8)
Fibrinogen, mg/dL	280.19 (140.7)
Ferritin, µg/L	13 069.12 (11 119)
PT, s	17.08 (5.6)
PTT, s	40.88 (11.6)
Neutrophils/µL	2256.15 (1481.5)
Hemoglobin, g/dL	7.19 (1.6)
Platelets/µL	48 076.92 (37 842)

Abbreviations: PT, prothrombin time; PTT, partial thromboplastin time; SD, standard deviation.

The comparative analysis between survivors and nonsurvivors of the HLH-2004 criteria determined that none of the variables had statistical significance for the mortality rate at 30 days. Laboratory parameters were compared between both groups, and PT and PTT differences were significantly different. For the PTT analysis, mean differences were calculated using Student *t* test (*P* = .01), and the Mann-Whitney test was used for PT (*P* = .004), since it’s distribution was non parametric (Table 3). After multivariate analysis using logistic regression, both variables lost statistical significance, with an adjusted odds ratio for PTT of 1.2 (95% confidence interval, 1–1.5; *P* = .05) and for PT of 1.4 (.92–2.36; *P* = .1).

**Table 3.** Comparison of Laboratory Results Between Survivors and Nonsurvivors at 30 Days

Laboratory Parameter	Value, Mean (SD)		Mean Difference (95% CI)	*P* Value^a^
	Survivors	Nonsurvivors		
Viral load, copies	2 892 394 (5 138 161)	2 838 376 (3 330 637)	NA	.40
CD4**^+^** T-cell count, cells/µL	26 (23)	26 (14)	NA	.75
Alanine aminotransferase, IU/L	59 (45.3)	86.11 (48.1)	NA	.18
Aspartate aminotransferase, IU/L	205.4 (259.42)	342.1 (365.3)	NA	.67
γ-Glutamyl transferase, IU/L	279.71 (214.8)	246.2 (138.3)	NA	.92
Alkaline phosphatase, IU/L	47 812 (327.6)	808 (732.3)	NA	.46
Lactic lactate dehydrogenase, IU/L	1250.53(1263.9)	3565 (4222.31)	NA [29]	.18
C-reactive protein, mg/L	190.71 (111.89)	222.56 (73.41)	NA	.15
Triglycerides, mg/dL	273.29 (173.51)	352.56 (347.82)	NA	.56
Fibrinogen, mg/dL	248.12 (97.51)	340.78 (191.14)	NA	.24
Ferritin, µg/L	12 193.4 (11 803)	14 723.2 (10 149.6)	NA	.40
PT, s	14.9 (3.1)	21.11 (7.13)	NA	.004
PTT, s	35.71 (5.8)	50.67 (13.8)	−14.9 (−25.7 to −4.14)	.01^b^
Hemoglobin, g/dL	7.18 (1.7)	7.22 (1.4)	NA	.96
Platelets/µL	38 294.1 (30 768.8)	66 555.5 (44 657.3)	NA	.10
Neutrophils/µL	1981 (997)	2775 (2098)	−794.38 (−2444.6 to 855.97)	.30^b^
HScore	248.77 (29.1)	249.08	NA	.95

Abbreviations: CI, confidence interval; NA, not applicable; PT, prothrombin time; PTT< partial thromboplastin time; SD, standard deviation.

^a^ *P* values calculated with Mann-Whitney *U* test unless otherwise specified.

^b^Calculated with Student *t* test.

### Associated Comorbid Conditions and Treatment

We also found that concomitant diseases were documented in 15 patients (57.6%), including probable pneumonia due to *Pneumocystis jirovecii* (PJP) (n = 7), IRIS (n = 4), and bacteremia due to *Salmonella* spp. (n = 4). Of these, 96.16% received targeted therapy for the triggers, except 1 with a postmortem diagnosis of disseminated histoplasmosis. Furthermore, 73% received amphotericin B deoxycholate; the remainder received itraconazole. ART initiation was proposed for the survivors until the acute disease subsided.

### Outcomes

Patients were followed up for a median (interquartile range) of 202 days (1079 days) with a 50% mortality rate at the time of data collection and a 30-day mortality rate of 34.6%.

## DISCUSSION

This retrospective review, conducted over 5 years. provides a description of the clinical and biochemical characteristics and the outcomes in patients with disseminated histoplasmosis and HIV/AIDS that presented with HLH. It is the largest series reported to date at a single center, which may be associated with the high prevalence of disseminated histoplasmosis in Latin America.

The distinctive characteristics of HLH are abnormal immune response and decreased levels or absence of NK cells and CD8^+^ cytotoxic T cells [12, 14]. Therefore, PLHIV are predisposed to this condition due to functional defects of NK cells and decreased CD4^+^ T- cell counts [12]. Mechanisms linked to the innate immune response, in these cases, favor the progression of disseminated histoplasmosis [14, 15].

Neoplasms and infections are frequently reported triggers [13, 16] in PLHIV. Another review found that disseminated histoplasmosis is a frequent cause of HLH [11], documented mainly in endemic areas. In our investigation, 36.1% of patients with disseminated histoplasmosis presented with HLH. This condition was more prevalent in men (84.9%) and young adults (80.7%), consistent with findings of previous studies conducted in Brazil and Peru [12, 13]. Furthermore, 100% of patients came from Latin American countries, which highlights the endemicity of this microorganism, now favored by migratory movement.

Half of the patients in our cohort were unaware of their HIV diagnosis at the time of HLH presentation. The other half knew about it but had virological failure due to poor adherence to ART. In this group, 15.4% had recent therapy onset or restart and presented possible unmasked IRIS, with a mean of 19.5 days since ART initiation. The presence of IRIS due to disseminated histoplasmosis is considered rare. A retrospective multicenter study was carried out in French Guiana from 1997 to 2017, where 22 cases were included, with a median of 11 days from the start of treatment [17]. It is unknown how many of them presented with HLH, and although it considered a rare complication, it is associated with high morbidity and mortality rates [18, 19].

In our current study, 100% of patients were in category 3 [29], according to the Centers for Disease Control and Prevention 2014 classification, with an mean CD4^+^ T-cell count (SD) of 26/µL (20.4/µL). This explains the presence of AIDS-defining diseases [18–20]. The diagnosis of HLH represents a clinical challenge and requires a high index of diagnostic suspicion. In some cases, diagnosis is based on positive tissue pathological findings since clear documentation of ≥5 of the 8 HLH-2004 criteria is not completed [21]. In our investigation, 100% of patients had an HScore >169, of whom 34.6% met 5 of the 8 HLH-2004 criteria. Hemophagocytosis was detected in only 7.6% of patients. This parameter, although suggestive, lacks sensitivity because its presence can be cyclical, and a sample can provide a false-negative result [2, 3, 10]. It is worth noting that HScore is a valuable tool for predicting diagnosis, and it is open source and easily accessible despite its limitations [10].

Disseminated histoplasmosis was confirmed by culture and/or urinary antigen testing. Results of histopathology were positive in only 5 patients, which limited the HLH prediction analysis. Antigen detection was not performed in 12 of 26 patients due to the unavailability of the test. This favors underdiagnosis since cultures have limited sensitivity and require more time and biosafety precautions. Therefore, we emphasize the importance of conducting urinary antigen detection for *H capsulatum* due to its high sensitivity [19, 20, 22–24].

The most frequent manifestations of HLH were hepatomegaly (100%), fever (96.2%), dyspnea (84.6%), and splenomegaly (76.9%). These results are consistent with those reported in the literature [25–27]. HLH has a wide spectrum of clinical manifestations, depending on its trigger, comorbid conditions, and severity. The involvement of tissues, bone marrow, liver, and central nervous system occurs mainly due to infiltration by T cells and macrophages via the secretion of cytokines and the initiation of phagocytic activity. Elevated levels of interleukin 1 and 6 and tumor necrosis factor α cause fever and hypertriglyceridemia by inhibiting lipoprotein lipase, and the secretion of activated macrophages causes high ferritin levels [1].

Regarding laboratory alterations, hyperferritinemia (100%), elevated lactate dehydrogenase (100%), and bicytopenia (61.5%) were common in both survivors and nonsurvivors. This is consistent with other research that determined the predominance of hyperferritinemia (100%) and bicytopenia (71.4%) [11]. A comparative analysis of risk factors for the 30-day mortality rate was conducted, with clinical and biochemical parameters that revealed the statistical significance for PT and PTT, which are not present in the HLH-2004 or HScore criteria. However, they are alterations suggestive of multiple organ dysfunction. These parameters are associated with mortality risk and may suggest disseminated intravascular coagulation, a complication associated with the mortality rate in critically ill patients with HLH [28]. Other studies need to be conducted to evaluate the presence of disseminated intravascular coagulation in PLHIV.

We found that 57.6% of patients had comorbid conditions, limiting the detection of the main secondary trigger of HLH. Cases of bacteremia due to *Salmonella* spp, probable PJP, and IRIS were found. PJP was not confirmed due to a lack of diagnostic tests, and we do not rule out the possibility that the lung involvement was secondary to disseminated histoplasmosis. Treatment was directed at the triggers, and none of the patients received immunotherapy for HLH because this indication is not clear in the context of disseminated histoplasmosis in PLHIV. World Health Organization guidelines suggest early initiation of antifungal treatment for disseminated histoplasmosis, since corticosteroids have a low level of evidence [18]. A systematic review found that steroid use was associated with higher mortality rate (*P* = .048) [28]. In our study, patients with probable PJP received systemic corticosteroids, which predominated in the group of nonsurvivors. These findings are likely related to each other; however, more research is required to provide close follow-up of subsequent clinical progress at the beginning of corticosteroid therapy.

The survival rate at 30 days was 65.4%. The overall mortality rate was 50% until the last day of data collection, similar to the 44% found in Brazil, and another study conducted in Peru found 50% [12, 13]. The main of death was septic shock and multiorgan dysfunction; none of the deaths had a postmortem report. Therefore, the combination of secondary triggers and laboratory abnormalities could help classify patients with HLH into different risk groups and guide timely treatment to reduce mortality rates. These results do not apply to other populations because the clinical presentation and severity of HLH depend on the triggers, degree of immunosuppression, and local epidemiology. Diagnosing and treating HLH in PLHIV continues to be challenging, and additional research is required for its characterization. The limitations of the current study are its retrospective design, small size, and the few patients undergoing urinary antigen testing for *H capsulatum* and histopathology tests.

In conclusion, we detected a high frequency of HLH in PLHIV with disseminated histoplasmosis. Therefore, we encourage physicians to suspect HLH in patients with fever, dyspnea, hepatosplenomegaly, cytopenias, hyperferritenia, and elevated lactate dehydrogenase levels, followed by the application of diagnostic prediction tools (HLH-2004 and HScore criteria), since early diagnosis and timely treatment reduces morbidity and mortality rates.
